# The Principles of Treatment in Pulmonary Tuberculosis, with Some Observations on Its Etiology

**Published:** 1889-06

**Authors:** Hermann M. Biggs

**Affiliations:** Pathologist to the New York City Health Department and Charity Hospital; Demonstrator of Anatomy in the Bellevue Hospital Medical College, and Instructor in the Carnegie Laboratory; Carnegie Laboratory, New York


Buffalo Medical and Surgical Journal

Vol. XXVIII. JUNE, 1889. No. 11.

### Original Communications.

### THE PRINCIPLES OF TREATMENT IN PULMONARY TUBERCULOSIS, WITH SOME OBSERVATIONS ON ITS ETIOLOGY.[1]

By HERMANN M. BIGGS, M. D.,

Pathologist to the New York City Health Department and Charity Hospital; Demonstrator of Anatomy in the Bellevue Hospital Medical College, and Instructor in the Carnegie Laboratory.

I SHALL offer no apology for having chosen for my paper before this Association a subject so much written upon as that of pulmonary tuberculosis; for, the prevention and treatment of tuberculosis seem to me subjects of such great importance, and so frequently regarded from an erroneous standpoint, that I feel more than justified in bringing them again before the profession.

The announcement by Koch, in 1882, of the discovery of the tubercle bacillus, and the demonstration by him of its essential etiological relation to this disease, marked the beginning of an important epoch in the history not only of this affection but also of the infectious diseases in general. His observations have compelled a complete remodeling and reconstruction of our conceptions of this disease. Nevertheless, many of even the more progressive men of the profession have not yet been able to relinquish them for the new, and will not accept proven facts; or, if theoretically these facts are accepted, their practical bearing is but imperfectly appreciated. The importance of this subject cannot

1. Read at the annual meeting of the Alumni Association, Niagara University, Medical Department, April 9, 1889.

be over-estimated, and I believe that the *great sanitary question of the future has to do with the prevention aud restriction of pulmonary tuberculosis.* From a practical sanitary standpoint, no disease compares with this in its vast significance; nearly one-seventh of all deaths are due to it, and about one-fourth of those occurring during adult life are caused by it.

Koch has said that "so soon as the fact is recognized by physicians generally, that tuberculosis is an exquisitely infectious disease, the means of attacking it must develop themselves."

So thoroughly now has the infectious nature of tuberculosis been established, that it is unnecessary in this paper to state the grounds upon which we assume that the tubercle bacillus is the *cause*, and the *only cause*, of tuberculosis in the human being and in animals. In support of this assumption we will only say that there is perfect unanimity of opinion among all workers in pathology and bacteriology upon this question, and that this statement is accepted without reservation. It is not a question of opinion, but it is a thoroughly demonstrated and demonstrable fact.

However, in the consideration of the etiology of tuberculosis, assuming that the tubercle bacillus is the sole direct cause, there are yet some questions to be studied of great importance; and the first of these, perhaps, is heredity. We may assume with absolute certainty that tuberculosis is only very rarely, if ever, transmitted *in utero.* There is no recorded case of intra-uterine tuberculosis in the human being, although Virchow is said to have seen such a case. One instance has been reported in the calf by Johne, and there are other facts tending strongly to show that the disease is sometimes hereditary in animals. Possibly—we may say, perhaps, that probably—in some exceedingly rare cases in the human being, where there is general tuberculosis, or, perhaps, a tuberculosis of the female genital organs, the disease may be transmitted to the fetus *in utero*, but these cases are too rare to be of much practical importance. In this connection it has been justly argued, and with much force, that if tuberculosis was often inherited, it would manifest itself more frequently in early and middle life, whereas, in reality, it increases regularly with advancing age. The suggestion thrown out by Councilman, that possibly tuberculosis sometimes exists at birth,—as in cases of primary joint and bone tuberculosis,—but produces lesions that are as yet not recognized at all, or not recognized as tubercular, does not seem to us probable, and has no confirmatory facts in its support. The practical point is, that in at least ninety-nine (99) per cent. of all cases of tuberculosis, the infection occurs after birth, and the disease is not transmitted by heredity. This fact cannot be too strongly emphasized, for, even if an inherited *predisposition* to the disease is presumed, which we do not at all admit, there is still a vast difference between an existing disease acquired by inheritance and a predisposition to a disease, development of which may be prevented by the observation of certain precautions. Further, the infectious nature of tuberculosis being admitted, and its transmission by heredity being excluded, the disease at once is thrown into the class of preventable diseases.

What, then, is the explanation of the constant occurrence of pulmonary tuberculosis in several members of the same family? We have no difficulty in accounting for the occurrence of several cases of scarlatina or diphtheria in families; these diseases are certainly transmitted from person to person. Not less certainly is tuberculosis so transmitted.

It has been frequently suggested that the occurrence of pulmonary tuberculosis in the children of tubercular parents is due to the transmission not of the disease itself but of a predisposition to the disease, viz., the transmission from the parents to the offspring not only of tissues with a resisting power that is below the normal, but of tissues that are also especially susceptible to the tubercular virus, so that when exposure to infection occurs, as it inevitably must occur, the tubercle bacilli find unusually fertile soil for their growth. This is true in only a very limited way. The offspring of delicate parents, whether they are tubercular or not, as a rule, are less robust than those of strong and healthy individuals. The children have less resisting power, and are more easily affected by the action of noxious influences of all kinds; this is certainly often true in the case of children of tubercular parents. There is, however, no special susceptibility that is transmitted, but simply a peculiar delicacy of constitution, that, as has been said, shows less resisting power when exposed to the action of any form of noxious influence, including the tubercle bacillus. It is the absence of strength, and not the possession of unfavorable qualities, that is transmitted. I think, however, the largest factor in the production of such series of cases in families is not this inherited delicacy of constitution, but it is their constant exposure to infection with the tubercle bacilli, under the most favorable conditions, from the earliest moments of life. With the existence of tuberculosis in the parents, so that *there is constant exposure* to infection, there almost certainly comes a time when from some other cause—perhaps poor or improper food, or disturbance of digestion, with mal-nutrition, or an attack of some acute infectious disease, such as measles or whooping-cough—the local or general resistance of the tissues of the child is so reduced, that the child becomes susceptible to the bacilli, which it from time to time is receiving from one or both of its parents. These may be received not only in the inspired air, but also in food, or in the milk from tubercular cows or nurses. The relations also usually obtaining between children and parents, and especially between children and their mother, are of that close and intimate nature especially favorable to the transmission of such disease by direct contact.

We may premise a little now in saying that not only are the discharges from cases of tuberculosis infectious, and the principal source of infection, but that they are the *only* source of infection. As to the avenues of infection, in by far the largest proportion of cases, infection takes place through the respiratory tract. Occasionally pulmonary tuberculosis is secondary to primary disease of some other organ, as the intestines, genito-urinary track, lymphatic system, or bones and joints; these cases, however, form a comparatively small proportion of the total number.

The question then arises, What are the products that contain the tubercle bacilli, and how do these become the means of infection? That the tubercle bacilli are present in large numbers in the sputum of most cases of pulmonary tuberculosis, has been abundantly proved; further, that they, or their spores, are present in all tubercular processes and in the discharges from these, has also been again and again demonstrated. The sputum and these discharges will produce tuberculosis in susceptible species of animals, when introduced in large enough quantity in any manner into the tissues of such animals. The entrance may be through the respiratory or alimentary tract, or by intra-peritoneal inoculation. The experiments to show that tuberculosis may be produced in animals by causing them to breathe air impregnated with dry and pulverized tubercular sputum, or atomized sputum, or by feeding them with matter containing tubercle bacilli, have been so numerous and so many times repeated, that it seems scarcely necessary to refer to them. The same is true of the intra-peritoneal inoculation of animals with such matter. The disease may be produced also quite as readily, in the same manner, by the substitution of cultivations of the tubercle bacilli for the sputum. A proof that the deductions drawn from the inhalation experiments on animals also hold true with regard to man, is afforded by the remarkable example reported by Schwenninger, of the possibility of infection of man when placed under the same conditions in which animals are infected, *i. e.* by impregnating the air with the tubercle bacillus. During the time that Tappeiner was conducting his inhalation experiments on animals, the servant who had charge of them, a very robust man of forty years of age, with absolutely no hereditary predisposition to the disease, and who had previously been perfectly healthy, notwithstanding the strong protestations and warnings not to enter, or remain, in the inhalation room, disregarded these directions in order to show that this procedure was comparatively free from danger, and acquired in this way the same form of inhalation tuberculosis as had been produced in the dogs experimented upon. He died from tuberculosis after about fourteen weeks, and the autopsy revealed the presence of the same lesions as had been previously found in dogs that had been killed,—only in him the disease was farther advanced, corresponding to the longer period of time. Further careful experiments have shown that the *air expired* by patients suffering from pulmonary tuberculosis does *not* contain the tubercle bacilli, and is not infectious. This is easily explained, in view of the abundant experimental proof that bacteria are not taken up from moist surfaces or from liquids containing them. The perspiration does not contain them, and if the discharges from the alimentary canal at any time do contain bacilli, there is little chance of infection from these, excepting through the possible contamination of the water supply. This is so improbable as to be thrown out of the question. There remains, then, as the only important source of infection, the discharges from the air passages—that is, the expectoration; from this arises by far the greatest danger. While the sputum is moist it is comparatively innocuous, unless taken in with the food. If, however, it has been deposited on the floor, carpets, clothing, furniture, handkerchiefs, etc., or, in fact, anywhere outside of the receptacle intended for it, it soon becomes dried, and then later, perhaps, pulverized, becomes suspended in the air, in the form of dust, and then is taken in with the inspired air. It has been shown experimentally that this is what constantly occurs. In this manner favorable opportunities for infection of the air passages are presented.

The cases of infection of human beings from tubercular animals, although probably more numerous than is generally supposed, yet form a comparatively small proportion of the whole number. We cannot at all accept the conclusions of Brush, that the larger proportion of the cases in the human being are due to infection from the bovine species. This mode of infection certainly accounts for but a small proportion of cases.

By far the largest number of cases of human tuberculosis are undoubtedly due to infection from other human beings affected with the disease. Some conception of the wide and general diffusion of the tubercle bacilli, where there are tubercular patients, may be gained from a review of the very careful and extensive experiments of George Cornet, published in the *Zeitschrift für Hygiene.* He collected dust from the most varied points, in hospital wards, insane asylums, prisons, clinic rooms, private houses, and so on, where phthisical patients were present, or had been present, and from others which had been occupied for a longer or shorter time, or not at all, by phthisical patients. The dust was taken from parts where the sputum of patients could not have well lodged when expectorated, viz., from the side walls, back of the patient's head, from the mouldings and high-hanging pictures, from under the beds, and from the cross-pieces of the bedstead near the head, from dishes placed in the room to collect the dust, etc. This dust was used for the inoculation of guinea-pigs, which are very susceptible to tuberculosis, although they rarely suffer from this disease in the natural state. Complete precautions were employed in the inoculations. The results obtained were in brief as follows: A large proportion of the animals inoculated with dust collected from the most varied points, in rooms of public institutions and private houses, where there were cases of tuberculosis, developed the disease; and, on the other hand, where the dust was taken from places where there were no cases of this disease, and had been none, the animals inoculated remained free. More than this, in rooms in private houses where there had been cases of phthisis, the dust was found to be virulent not only during the occupancy by phthisical patients but in some instances for a number of weeks after the removal of the patients.

Although these observations simply place on a sound experimental basis the opinions previously held by many pathologists, yet their important practical bearing is at once perceived. They imply that where there are cases of tuberculosis under the usual conditions, the dust surrounding them often contains tubercle bacilli, and persons inhaling the air in which this dust is suspended must, from time to time, inhale tubercle bacilli.

As we may exclude from our consideration for the present those cases where the disease results from infection through other channels than the air passages, we may sum up the whole question regarding this aspect of the etiology of this disease in a few words. Tuberculosis is not inherited—it is always the result of the action of tubercle bacilli. It has been abundantly proved by Koch that the tubercle bacilli cannot multiply outside of a living organism, human or animal (except when cultivated artificially); hence it follows, and there is no way of avoiding this conclusion, that when tuberculosis occurs in the animal or human organism, it is the result of infection by tubercle bacilli, derived directly from some other animal or human being affected with tuberculosis. When we remember what enormous numbers of the bacillus tuberculosis are present in the sputum, and what large quantities of expectoration are sometimes discharged for months and years by patients affected with chronic pulmonary tuberculosis, and that the bacilli, or their spores, retain there virulence for weeks and months after these discharges, we can readily understand that the opportunities and sources for infection with the tubercle bacilli may be constantly presented.

This brings us to the consideration of another phase of the subject. An almost casual examination of the observed facts at once arouses the conviction that, under natural conditions, something more than the mere presence of the tubercle bacilli is ordinarily necessary to produce pulmonary tuberculosis; for, so general is tuberculosis, that under the conditions found in modern civilized countries, every individual who has attained maturity must have been exposed at some time in life.

Tuberculosis is originally always a local, infectious disease; preceding the development of the local process there is usually, and perhaps always, a depressed condition of general vitality, or of the local vitality of the organs or tissues affected, which renders the individual susceptible to the disease. The relation of this local or general condition to the tubercular process, and its influence upon prevention and treatment, will be better understood after referring briefly to some points in regard to the transmission of the infectious diseases. It has been shown experimentally that all individuals, however susceptible the species or individual may be, yet possess a certain degree of immunity to diseases to which they may be subject; in other words, there is a definite relation between the specific virus, causing some infectious diseases (and probably this is true of all), and the tissue resistance of the individual. This insusceptibility varies with different individuals, and with the same individual at different times, and is largely dependent upon the intensity of the virulence of the virus employed, the amount of virus used, and the condition and surroundings of the individual experimented upon.

[1] It has been found, in the case of certain of these diseases, that the other conditions being the same, the effect produced is directly proportionate to the amount of the virus used. If a very small amount is introduced subcutaneously, no effect follows the inoculation; if a somewhat larger amount is introduced, a local disturbance at the point of inoculation follows; and if a still larger amount is used, the characteristic phenomena of the disease are produced. In other words, the natural resistance of the individual to the disease in question must be first overcome by the size of the dose of the virus. We may represent this graphically by supposing that $x =$ the normal resistance of an individual to a given disease, and $y =$ amount of virus sufficient to produce the disease. These are both variable quantities; $x$ varies with the individual and with time, condition, etc.; $y$ varies with the individual susceptibility and with the intensity of the virus. It will be readily seen that immunity or increased susceptibility, or their equivalents, may be produced by variations in either $x$ or $y$. The insusceptibility to any disease may be so great that under natural conditions the intensity of exposure to infection, or, in other words, the dose of the virus received, is not great enough to overcome this insusceptibility, and such individuals possess practically absolute immunity to the disease. Some persons have such an immunity to small-pox; but there can be no doubt that in such individuals this immunity could be overcome artificially, if it were possible to increase indefinitely

1. Biggs in Wood's Reference Hand-Book of Medical Sciences.

the dose of the virus. Now, in a local parasitic disease, such as tuberculosis originally is, it becomes evident at once that any influence which diminishes the resistance of any organ or tissue that is exposed to infection, whether this influence is a local or general one, by just so much contributes to the production of the disease.

The depreciated condition of general health or lack of tissue resistance that usually precedes the development of tuberculosis, may be congenital or acquired, and it is because of the frequent development of tuberculosis, in some form, in those persons who have had transmitted to them at birth this condition, *i. e.*, where there is a weak resisting power, that the disease has been so long considered hereditary.

But aside from this general condition of depressed vitality, that so often precedes the development of this disease, there may also be produced by various causes a local depression of vitality in some tissues exposed to infection that has the same effect. It is the combination of these two sets of conditions, general and local, that offers the best opportunities for the development of the tubercle bacillus. As an example of the development of tuberculosis under such conditions, we may instance the occurrence of tubercular joint disease in children after traumatism, when the depreciated state of general health preëxists. The traumatism produces the local depression, which determines the site of the tubercular process.

It may be noted here that those diseases which are associated with some erosions of the mucous membrane of the smaller bronchial tubes, accompanied as these are with destruction of the ciliated epithelium covering the membrane, probably more strongly predispose to the development of pulmonary tuberculosis, than those affections which produce a general depression of vitality without any local disorder of this nature. Such eroded spots offer favorable points for the lodgment and development of the tubercle bacilli. The very frequent occurrence of this disease after measles and whooping-cough, is probably to be accounted for in this way, and not because of their producing any special or peculiar susceptibility to the disease.

The tubercle bacillus is the active cause of the disease. If introduced in large enough numbers, this will produce the disease independently of any other influence; but, under natural conditions, if no local or general depression of nutrition exists, the intensity of exposure to infection, or the number of tubercle bacilli received at any given time, is not sufficient to overcome the natural insusceptibility. When, however, the natural resisting power to the disease is lessened from any cause, the dose of tubercle bacilli that before proved insufficient, now results in the production of the disease.

Further than this, the same conditions exist later on, if the tissues possess a certain degree of vigor, and if the standard of nutrition is brought up to certain level, they resist the multiplication of the tubercle bacilli and the extension of the tubercular process; this then becomes stationary or retrogressive. If, on the other hand, the vitality of the tissues is low and the conditions are favorable for the development of the tubercle bacilli, they find little resistance to their multiplication, and the disease rapidly extends. That this is exactly what occurs, is shown clearly enough by the conditions found after death. In more than sixty (60) per cent. of the autopsies in the charity hospitals of New York, I have found, after death, in the lungs the evidence of a tubercular process that had existed at some time in life. This was manifested in the form of cheesy or calcareous masses, fibroid tubercles, or well-marked tubercular lesions. This observation conveys some conception of the alarming frequency with which this disease prevails, and how large a proportion of the human race become infected at some time in life. There is, however, another side,— a bright side,— brought out by these facts, which shows in the most unmistakable manner that tuberculosis does not tend to end fatally in all or in most cases; that it is not invariably an incurable disease, in the ordinary acceptation of that term; but, on the other hand, that in a very large proportion of cases the process early becomes stationary and retrogressive. I

have found that this was the case in nearly fifty (50) per cent. of the instances of pulmonary tuberculosis, and that in every stage of the disease, up to the time when there were cavities in both lungs, with a large proportion of the upper lobe of the one lung converted into cavities, that the process may become stationary or retrogressive. The cavities cicatrize, or become surrounded by fibrous tissue walls; the tubercular masses become cheesy, or calcareous, and surrounded by a fibrous tissue capsule. We must bear in mind, in this connection, that the disease is no less pulmonary tuberculosis, if there is a single nodule of a tubercular nature in one lung, than if both lungs are completely filled with tubercular lesions. In a large proportion of the cases where the disease became stationary, a small amount only of lung tissue had been involved, but in a few instances, as I have said, quite the reverse of this was the case. In many of these subjects the individuals had probably never been conscious of the existence of any serious pulmonary disease, but, at some time in life, as the result of some depressing influences, conditions favorable to the development of tubercle bacilli had been produced, the subjects had been exposed to infection, and a local tuberculosis had resulted. Later, the conditions had changed, the local or general state of nutrition had improved, and with this came a corresponding increase in the resisting power of the tissues; they no longer afforded favorable soil for the growth of the germs, and the disease early ceased to extend. These observations have an important, practical bearing, for they show, in the most unmistakable manner, that in a large proportion of cases pulmonary tuberculosis tends to become self-limited, and that it is distinctly amenable to treatment. It only tends to end fatally of necessity where the amount of involvement of lung tissue is great.

The environment experiments of Trudeau bear out the conception of this disease just presented, in the most complete manner. He found that if rabbits were inoculated with cultures of the tubercle bacilli, and were then placed under the most favorable conditions of life, they did not ordinarily develop the disease; but other animals, inoculated at the same time under precisely the same conditions, invariably developed the disease when placed under unfavorable conditions of life. The favorable surroundings in the one case afforded the animal a greater resistance and consequent immunity to such doses of the virus; but unfavorable surroundings in the others rendered the animals susceptible to the disease. These considerations give us the key to the whole subject of the treatment of tuberculosis.

For, keeping in mind these two etiological factors—first, the tubercle bacillus, and second, the conditions that render the tissues a favorable site for their development—the principles of treatment become at once clearly defined.

As regards the tubercle bacillus, it becomes at once apparent that in all forms of internal tuberculosis, when the diseased tissue cannot be removed surgically, treatment directed specially toward the parasite (i. e., germicidal treatment in the ordinary sense) is futile. From the nature of the pathological changes that occur, and from the position of the bacillus in the diseased tissues, it becomes evident that no germicidal agent can be brought directly in contact with the organism, and we may dismiss at once from our thought any idea of accomplishing any direct result by any form of antiseptic or germicidal treatment, whether it comes from the internal administration of germicidal agents, or antiseptic sprays or inhalations, or injections, or gaseous enemata. Any apparent results that are obtained by these means are indirect at best. They divert our attention from the only rational line of treatment, and fall far short of accomplishing the object for which they are intended. They have not one single physiological or pathological fact for their basis, and are founded on a kind of a sentimental system of bacteriological empiricism. It seems to me simply deplorable that such a method of treatment as that of rectal injections of sulphurated hydrogen should have received, for a time, such wide recognition by the profession in these days of scientific medicine.

If we look to the second factor in causation, viz., diminished tissue resistance, the prospect becomes at once bright. We find the treatment based upon known facts in regard to the pathological anatomy and pathogenesis of this disease, to be in perfect accord with the deductions drawn from clinical experience. Any method of treatment that tends to improve the condition of general nutrition also tends, by just so much as it increases the resisting power of the tissues, to limit and check the extension of the disease. Further, any method of treatment that raises the local vigor of the organs or tissues affected, tends in the same way to increase the resisting power of the tissues, and so tends to check and limit the extension of the tubercular process. All medicinal methods of treatment that have withstood a long and severe test of clinical experience, come under one of these heads.

The treatment is thus narrowed down to those measures, for the most part hygienic in nature, which aim to bring up the standard of general nutrition to the highest possible point. In conjunction with such means may be sometimes used those that influence in the same way especially the vitality of the organ affected.

From this standpoint, inasmuch as the susceptibility may be inherited or acquired, the question of prophylaxis is a most important one; for, it is manifest that the chances of the patient susceptible to tuberculosis are vastly better before than after he has become infected with the exciting cause of tubercular changes. If we are dealing in any case with an inherited or acquired susceptibility, the object of prophylaxis is to place the patient in surroundings free from infective matter, in an a-tubercular atmosphere, and then to remove, if possible, the diathesis. As regards the prevention of infection: Recognizing the many avenues of infection and the fact that, although it is certain that the parasite does not live any stage of its existence outside of the body of animals, yet the spores are capable of withstanding successfully, for a considerable time, conditions averse to their development; recognizing these facts, the greatest consideration must be given to the following points: The sputum of phthisical patients should be at all times thoroughly disinfected, germicidal solutions in glycerine and water should be constantly standing in the receptacles for expectoration. All handkerchiefs contaminated with such infective matter should be placed in a solution of carbolic acid (1-20) before being washed. All cloths and rags so contaminated should be burned, and all clothes containing infective matter from tubercular ulcers of skin, or intestinal discharges from cases of tubercular enteritis, etc., should be treated in like manner. The attendant should not occupy the same bed as his patient, nor should he live continuously in one room if it can be possibly avoided. In the case of husband and wife, this rule should certainly be enforced. There is strong evidence to support the belief that tuberculosis is sometimes communicated *per coitus* through the genital organs. Infants should not be suckled by tuberculous nurses, nor should they sleep with tuberculous parents. Rooms occupied constantly by tubercular patients should be frequently thoroughly cleaned and disinfected.

To remove the susceptibility, and to limit the extension of the tubercular process when once established, the measures of treatment may be considered under the heads: Atmosphere and climate, food, exercise and occupation, baths and drugs.

These subjects it is not necessary to consider in detail.

As to the prevention of those cases due to infection of human beings from animals, it is clear that there is but one method, viz., governmental inspection of dairy cows and of animals slaughtered for food, the destruction of all tubercular animals, and the rigid exclusion of the meat for purposes of food. Brush suggests that the use of tubercular animals for breeding purposes, and the in-and-in breeding of any of the bovine species (which especially tends to develop tuberculosis), be prohibited by law. To show the frequency of the disease in cattle, it may be noted that competent and conservative veterinarians claim that from ten to fourteen per cent. of the thoroughbred Jerseys are affected with tuberculosis.

The points to which I had especially desired to call your attention are: That the question of the prevention and restriction of tuberculosis is of vast importance; that tuberculosis is a purely infectious disease; that it is not frequently transmitted by heredity; that it belongs to the class of strictly preventable diseases; that the sputum is the chief agent of transmission of the disease; and that, consequently, great care should be exercised in its disposition. Further, that under natural conditions of exposure to infection, there is usually necessary some inherited or acquired reduction of tissue resistance,—which is the result, as a rule, of a low standard of general nutrition; that the disease does not always tend to end fatally, but that, on the contrary, in at least fifty per cent. of the cases of pulmonary tuberculosis, the disease early becomes stationary and retrogressive; and, finally, that the only rational measures of treatment, so far as the tubercular process itself is concerned, are those that tend to raise the standard of general nutrition to the highest possible point, and so help to increase tissue resistance.

Carnegie Laboratory, New York.

---

The Buffalo Medical and Surgical Association held its regular monthly meeting May 7, 1889. Drs. George N. Burwell and J. B. Samo, the only surviving founders of the Association, sat with the President on this occasion, and gave some reminiscences of the early history of the Association.

Dr. Hartwig read a paper on Sterility in the Female.

The next meeting will be held at the Mechanics' Institute, No. 9 W. Mohawk street, June 4, 1889, at 8.30 p. m. Subject for discussion: In View of the Various Causes of Insanity, do the Insane Receive the best Medical and Surgical Treatment under the Present System of Asylum Management? If not, How can such Treatment be Secured?

Drs. Tremaine, Crego, Hopkins, Hurd and Putnam are expected to participate.

